# Early oral feeding following intestinal anastomosis surgery in infants: a multicenter real world study

**DOI:** 10.3389/fnut.2023.1185876

**Published:** 2023-07-20

**Authors:** Changgui Lu, Xinhe Sun, Qiming Geng, Weibing Tang

**Affiliations:** ^1^Department of Pediatric Surgery, Children’s Hospital of Nanjing Medical University, Nanjing, China; ^2^Nanjing Medical University, Nanjing, China

**Keywords:** early oral feeding, infant, intestinal anastomosis, safety, efficacy

## Abstract

**Background:**

To prevent postoperative complications, delayed oral feeding (DOF) remains a common model of care following pediatric intestinal anastomosis surgery; however, early oral feeding (EOF) has been shown to be safe and effective in reducing the incidence of complications and fast recovery after pediatric surgery. Unfortunately, the evidence in support of EOF after intestinal anastomosis (IA) in infants is insufficient. Therefore, this study was primarily designed to evaluate the safety and efficacy of EOF. In addition, the current status of EOF application and associated factors that favor or deter EOF implementation were also assessed.

**Methods:**

A total of 898 infants were divided into two groups (EOF group, *n* = 182; DOF group, *n* = 716), and the clinical characteristics were collected to identify the factors associated with EOF in infants. Complications and recovery were also compared to define the safety and efficacy after balancing the baseline data by propensity score matching (PSM) (EOF group, *n* = 179; DOF group, *n* = 319).

**Results:**

The total EOF rate in infants with IA was 20.3%. Multivariate logistic regression revealed significant differences in the EOF rates based on IA site and weight at the time of surgery (OR = 0.652, 95% CI: 0.542–0.784, *p* < 0.001) and (OR = 1.188, 95% CI: 1.036–1.362, *p* = 0.013), respectively. The duration of total parenteral nutrition (TPN), parenteral nutrition (PN), and postoperative hospital stay were significantly shorter in the EOF group than the DOF group [2.0 (1.0, 2.0) d vs. 5.0 (3.0, 6.0) d; 6.0 (5.0, 8.0) d vs. 8.0 (6.0, 11.0) d; 10.0 (7.0, 14.0) d vs. 12.0 (9.0, 15.0) d, all *p* < 0.001]. The rates of abdominal distension and vomiting in the EOF group were significantly higher than the DOF group (17.9% vs. 7.2%, *p* < 0.001; 7.8% vs. 2.5%, *p* = 0.006); however, no differences were found in failure to initial OF, diarrhea, hematochezia, and anastomotic leakage between the two groups (*p* > 0.05).

**Conclusion:**

The overall rate of EOF in infants following IA was low, and the sites of anastomosis and weight at surgery were two factors associated with EOF. Nevertheless, performing EOF in infants after IA was safe and effective, reduced PN usage, shortened the hospital stay, and did not increase the rate of severe complications.

**Clinical Trial Registration**: ClinicalTrails.gov, identifier NCT04464057.

## 1. Introduction

### 1.1. What is known

1. EOF has been shown to be safe and effective in reducing the incidence of complications and fast recovery after pediatric surgery; however, evidence for initiating EOF after an intestinal anastomosis in infants was insufficient.

2. Prolonged postoperative fasting or delayed oral feeding (DOF) remained a common and “traditional” model of care following pediatric intestinal anastomosis surgery to prevent postoperative nausea, vomiting, and anastomotic complications.

### 1.2. What is new

1. The current status of EOF in infants who underwent an intestinal anastomosis was evaluated and the associated factors with EOF were identified using multivariate logistic regression.

2. The safety and efficacy of EOF in infants who underwent an intestinal anastomosis were assessed using propensity score matching balancing baseline data.

Early enteral feeding (EEF), as a standard protocol for enhanced recovery after surgery (ERAS), has been shown to be safe and effective in some types of pediatric surgical procedures, is associated with a lower or similar overall incidence of complications, and promotes early bowel recovery and hospital discharge compared to delayed enteral feeding (DEF) (1–5). Prolonged postoperative fasting or delayed oral feeding (DOF) remains a common and “traditional” model of care following pediatric intestinal anastomosis (IA) procedures to prevent postoperative nausea, vomiting, and anastomotic complications. The DOF protocol was implemented based on the notion that anastomotic or ostomy healing is best achieved through bowel rest and reducing the risk of complications, such as anastomotic leakage or postoperative ileus (6, 7). In contrast, reports (8–10) have shown that withholding oral feeding does not eliminate 1–2 liters of endogenous fluids that would pass through the anastomosis, and bowel activity also occurs before the passage of flatus. Several animal studies (11–13) have also shown that prolonged fasting reduces the collagen content in anastomotic tissue and diminishes the quality of healing; however, early oral feeding (EOF) increases collagen deposition and the strength of the anastomosis site. Of note, EOF has been initiated in some elective pediatric IA procedures (6, 14, 15) and has been shown to be safe and effective, with a low incidence of complications and fast recovery after surgery. Nevertheless, further well-designed or multicenter studies are warranted to validate the safety and efficacy of EOF in pediatric IA procedures (7, 16).

The spectrum of diseases affecting infants is significantly different from that of other childhood age groups (17). Indeed, most malformations, particularly intestinal malformations, require surgical correction in infants. Due to the possibility of bowel movement dysfunction after correction of intestinal malformations, the route of early enteral feeding is usually via nasogastric or nasojejunal tubes (17, 18), which are dissimilar from normal physiology and can lead to many complications, including patient discomfort, tube malpositioning, aspiration pneumonia, sinusitis, epistaxis, and tube occlusion (19, 20). Because the body grows and develops more rapidly during infancy than in other periods during childhood, there are additional issues with postponing oral or enteral feeding, including cholestasis, sepsis, delayed gut development, and metabolic diseases (21–23). Unfortunately, evidence for initiating EOF after an IA procedure in infants is insufficient (24). Therefore, the current study was primarily designed to evaluate the safety and efficacy of EOF during the postoperative period among infants undergoing an IA, as well as the status of EOF application and its associated factors that favor or deter EOF implementation.

## 2. Methods

### 2.1. Study design and patients

A prospective multicenter study was conducted to evaluate the safety and efficacy of EOF in infants undergoing an IA between January and December 2021. The inclusion criteria were as follows: (1) patient with an IA; and (2) age at surgery < 1 year. The exclusion criteria were as follows: (1) preterm neonates with gestational age < 32 weeks or with a weight < 1.5 kg; (2) patients with a severe abdominal infection, intestinal perforation, meconium peritonitis, and severe intestinal adhesive disease; (3) significant difference in the proximal intestinal diameter and distal bowel diameter of the anastomosis impairing bowel movement [the proximal intestinal diameter and distal bowel diameter of the anastomosis ratio > 4: 1] (25), such as jejunal atresia; (4) patients with intestinal neuronal malformations or congenital intestinal motility disorders; and (5) patients initiated enteral feeding by a tube. A total of 898 infants who underwent an IA at 51 hospitals in mainland China were enrolled.

This study was approved and supervised by the Institutional Ethics Committee of the Children’s Hospital of Nanjing Medical University (approval number: 202004018-1) and registered at Clinical Trials.gov (NCT04464057). Written informed consent was obtained from the parents of all patients before enrollment in the study. The authors confirmed that all methods were carried out under relevant guidelines and regulations, and adhered to all research ethical guidelines of their clinical discipline, particularly where human or animal subjects were involved.

### 2.2. Oral feeding strategy

Oral feeding time was determined by each center according to usual practices without compulsory regulation and with no requirement for feeding nutrients. This study was based on the actual status of oral feeding after the IA and did not affect the actual behavior of the participating surgeons; however, the initial feeding amount of 10 mL/kg/d divided equally among 8 feeding times was usually in the EOF and DOF groups. If the patients tolerated feeding, the daily feed amount was increased daily (10–20 mL/kg). Feeding was suspended if vomiting occurred greater than three times daily, or if severe abdominal distention without passage of gas and stool by anus, or if bloating or bloody stools occurred until the symptoms resolved. The patients with suspended OF were defined as failure to initial OF, and the rates of failure to initial OF were recorded in EOF and DOF groups. Full OF was defined as 120 mL/kg/d without parenteral nutrition (PN) (24).

### 2.3. Statistical indicators and groups

**Figure 1:**
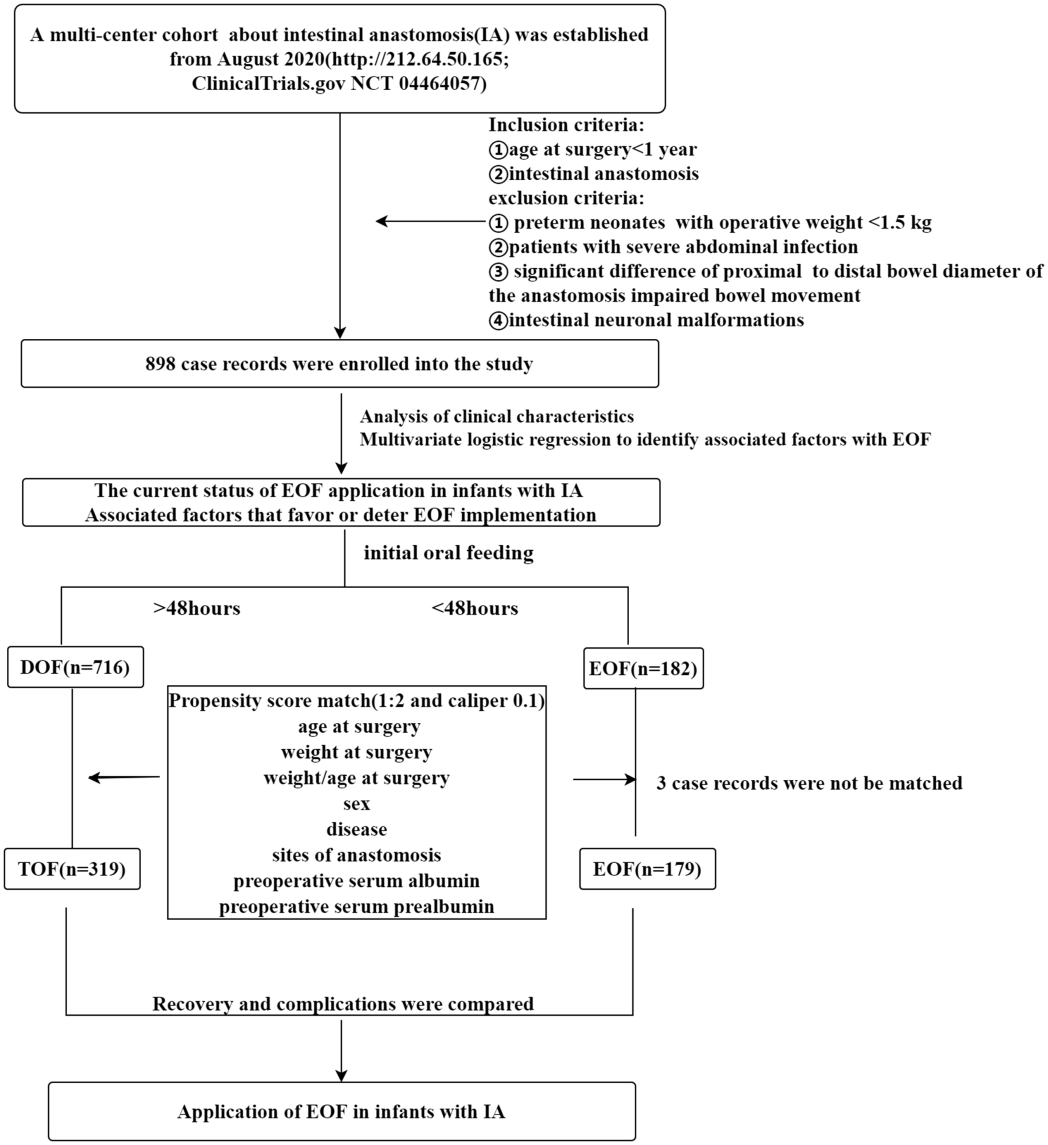
The flow chart of the study.

The age at surgery, weight at surgery, weight-to-age ratio at surgery, disease diagnosis, laparoscopic-assistance or not, initial feeding time, serum albumin and prealbumin levels before surgery, and sites of anastomosis were recorded as clinical characteristics. The occurrence of vomiting, abdominal distention, diarrhea, hematochezia, anastomotic leakage, failure to initial OF, PN time, hospital stay, weight at the time of hospital discharge, serum albumin level at the time of hospital discharge, prealbumin level at the time of hospital discharge, and the weight-to-age ratio at the time of surgery were also recorded to evaluate complications and recovery after EOF. After the data were collected, the patients were divided into two groups (EOF and DOF) according to the initial oral feeding time after the IA was performed. The EOF group (26) was defined as oral feeding starting within 48 h after surgery (*n* = 182) according to the recommendations of the American Nutrition Association (26–28). The DOF group (*n* = 716) was defined as oral feeding that started >48 h after surgery (Figure 1).

The status of EOF in the infants was evaluated after group assignment and the clinical characteristics were compared between the two groups to assess the baseline characteristics and identify the factors associated with EOF. The rates of complications and indicators of recovery were also compared between the two groups to evaluate the safety and efficacy of EOF after using PSM to balance the baseline between EOF and DOF.

### 2.4. Identification and management of confounding factors between the EOF and DOF groups

Because this study was an observational and real-world study rather than a randomized controlled study, there may have been significant confounding factors between EOF and DOF that affected outcome variables based on the relationship between confounding and exposure factors (in this study, receiving EOF) or outcome variables (in this study, complications and postoperative recovery). The confounding factors in this study were mainly those factors that affected EOF grouping, outcome variables, and both EOF grouping and outcome variables (29). There is currently no clear clinical indicator for EOF; however, the European Society for Parenteral and Enteral Nutrition (ESPEN) recommends assessing nutritional status preoperatively that may affect postoperative recovery (26, 28). In the current study, surgical weight, surgical age, surgical weight-to-age ratio, sex, serum albumin level, and prealbumin level before surgery indicated that nutritional status were confounding factors affecting outcome variables, and that OF should be initiated within hours after surgery in most patients according to individual tolerance and the type of surgery carried out with special caution (26). EOF has been reported in children with abdominal disease, gastrointestinal malformations, and children who have undergone a colorectal anastomosis (9, 14, 24). In previous study, the application of EOF for different diseases and anastomosis sites were evaluated (1, 30, 31), suggesting that the disease diagnosis and the anastomosis site may be important confounding factors for outcome. In addition, laparoscopic-assisted procedures reduced stress and intestinal adhesion formation, which may facilitate early recovery and early feeding after surgery (5). Specifically, nine covariates that influenced outcomes were defined and propensity scores were calculated, including surgical weight, surgical age, surgical weight-to-age ratio, sex, serum albumin level, prealbumin level, disease diagnosis, anastomosis site, and laparoscopic-assistance or not.

### 2.5. Statistical analysis

Data are presented as percentages, medians, or averages, and the level of statistical significance was set at 0.05. SPSS 22.0 was used for statistical analysis, and the distribution of continuous variables was examined for normality. A *t*-test was applied for normally-distributed data and a rank-sum test was performed for non-normally distributed data. Categorical variables were tested using the chi-squared or Fisher’s exact test, and the Bonferroni method was used to adjust the *p*-value in multiple group comparisons. Before comparing the complications and recovery between the EOF and DOF groups, a 1: 2 PSM with a caliper of 0.1 was applied to balance the baseline data between the two groups.

## 3. Results

### 3.1. Analysis of EOF status and factors associated with EOF in infants who underwent an IA

A total of 898 infants who underwent an IA in mainland China were recruited for this study, including 70 patients with intestinal atresia, 58 with a Meckel diverticulum or intestinal duplication, 52 with intestinal necrosis, 51 with intestinal stenosis, 209 with biliary atresia or a choledochal cyst, 343 with a stoma closure, 20 with a patent vitelline duct, 84 with duodenal obstruction, and 11 with intestinal adhesion-associated obstruction. The overall EOF rate in infants who underwent IA was 20.3%. Univariate analysis revealed no differences in the sex ratio, laparoscopy-assisted status, and weight-to-age ratio between the EOF and DOF groups (all *p* > 0.05). There was a significant difference in the EOF rate among the different diseases (*p* = 0.021), while the EOF rate for biliary disease was higher than intestinal atrophy (*p* = 0.001). A significant difference was also found at different anastomosis sites (*p* = 0.001), while the EOF rates for jejunal and colonic anastomoses were higher than ileal-colonic anastomoses (all *p* < 0.001). The weight at the time of surgery, age at the time of surgery, and preoperative albumin and prealbumin levels were significantly higher in the EOF group than the DOF group (all *p* < 0.05; Table 1).

**Table 1:** Baseline clinical characteristics of infants with intestinal anastomosis in EOF and DOF groups.

Group		EOF (*n* = 182)	DOF (*n* = 716)	*p*
*Diseases	Intestinal atresia (*n* = 70)	6	64	0.021
	Intestinal mass (*n* = 58)	11	47	
	Intestinal necrosis (*n* = 52)	11	41	
	Intestinal stenosis (*n* = 51)	7	44	
	Biliary disease (*n* = 209)	59	150	
	Closing the stoma (*n* = 343)	65	278	
	Patent vitelline duct (*n* = 20)	6	14	
	Duodenal obstruction (*n* = 84)	16	68	
	Adhesional obstruction (*n* = 11)	1	10	
**Sites of anastomosis	Duodenal anastomosis (*n* = 83)	16	67	0.001
	Jejunal anastomosis (*n* = 254)	66	188	
	Ileal anastomosis (*n* = 298)	52	246	
	Colonic anastomosis (*n* = 143)	37	106	
	Ileal-colonic anastomosis (*n* = 120)	11	109	
Laparoscopic assisted (yes/no, *n*)		36/146	148/568	0.790
Sex (male/female, *n*)		104/78	405/311	0.888
**Age at surgery (d, Median [P25%, P75%])		93.0 [41.3, 197.8]	76.0 [27.3, 140.5]	0.001
**Weight at surgery (kg, Median [P25%, P75%])		5.3 [4.3, 7.1]	4.6 [3.3, 6.0]	<0.001
Weight/age at surgery (g/d, Median [P25%, P75%])		58.9 [34.8, 109.7]	64.7 [41.2, 130.0]	0.056
*Preoperative albumin (g/L, Median [P25%, P75%])		39.8 [36.0, 43.0]	38.5 [34.8, 41.8]	0.007
*Preoperative prealbumin (g/L, Median [P25%, P75%])		0.16 [0.11, 0.17]	0.14 [0.10, 0.17]	0.027

Mann-Whitney U tests and Pearson’s chi square tests were used. *p < 0.05, **p < 0.001.

**Figure 2:**
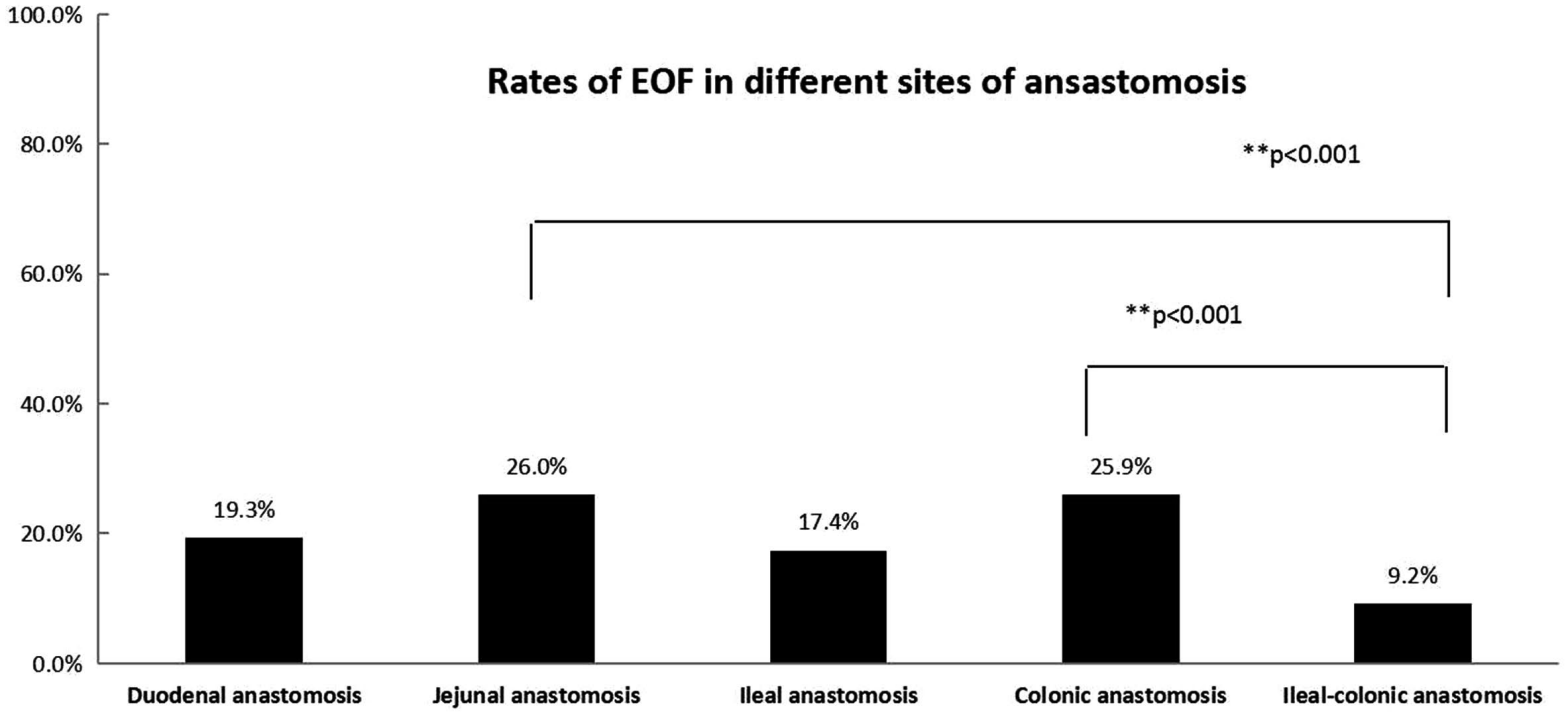
The rates of EOF in five types of anastomosis (duodenal, jejunal, ileal, colonic, and ileal-colonic) were significantly different (*p* < 0.05). The Bonferroni method was used to adjust the *p* value in multiple group comparisons. ^**^The rates of EOF in jejunal and colonic anastomoses were higher than ileal-colonic anastomosis.

**Figure 3:**
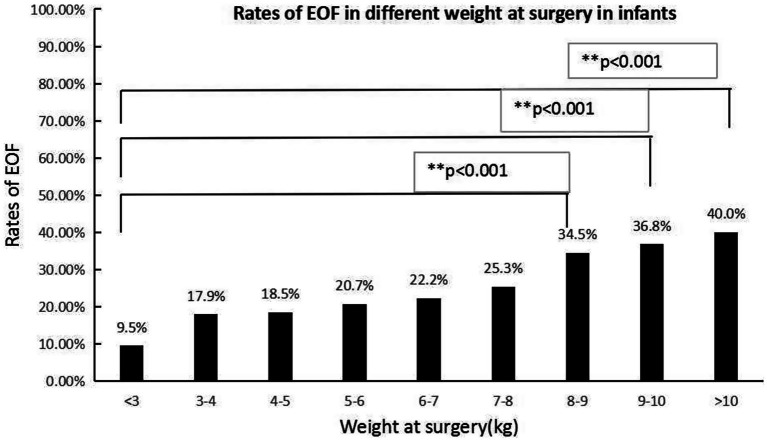
The EOF rates in various weights at the time of surgery were significantly different (*p* < 0.05). The Bonferroni method was used to adjust the *p* value in multiple group comparisons. ^**^The EOF rates in patients with a surgical weight >8 kg were higher than infants with a surgical weight <3 kg.

To avoid missing factors, the level of statistical significance for multivariate analysis was set at 0.1 and entered into a multivariate regression analysis to detect the real associated risk factors with EOF in infants who underwent an IA. The sites of anastomosis and weight at surgery were the only two factors associated with EOF in infants who underwent IA (OR = 0.652, 95% CI: 0.5420.652, 95% CI: a) and (OR = 1.188, 95% CI: 1.0368, 95% CI: CI: a), respectively; however, the disease diagnoses, age at surgery, and preoperative albumin and prealbumin levels did not exhibit differences after multivariate regression analysis (all *p* > 0.05; Table 2, Figures 2, 3).

**Table 2:** Multivariate logistic regression to identify associated factors with EOF in infants with IA.

Factors	*B*	SE	Wald	*p*	OR	95%CI
Diseases	0.018	0.051	0.123	0.725	1.018	0.921~1.125
**Sites of anastomosis	−0.428	0.094	20.668	<0.001	0.652	0.542~0.784
Age at surgery	0.001	0.002	0.781	0.377	1.001	0.998~1.005
*Weight at surgery	0.173	0.070	6.117	0.013	1.188	1.036~1.362
Weight/age at surgery	<0.001	<0.001	2.537	0.111	1.000	0.999~1.000
Pre-operative albumin	0.021	0.018	1.472	0.225	1.022	0.987~1.058
Pre-operative prealbumin	−0.264	1.270	0.043	0.836	0.768	0.064~9.265

B is the logistic regression coefficient and SE is the standard error. OR represents the odds ratio and 95% CI represents the 95% confidence interval. *p < 0.05, **p < 0.001.

### 3.2. Baseline data or associated factors (confounding factors) after PSM in infants who underwent IA

As a result of major imbalances between the EOF and DOF groups with respect to baseline data or associated factors, an approximate 1: 2 PSM with caliper 0.1 was used to balance the bias. Lastly, 179 EOF and 319 DOF records were successfully matched, and there were no differences in distribution of diseases, anastomotic sites, procedures with or without laparoscopic assistance, weight at the time of surgery, age at the time of surgery, the weight-to-age ratio, the serum albumin level, and the serum prealbumin level between the EOF and DOF groups (all *p* > 0.05; Table 3). Three records (biliary disease) in the EOF group were not matched, and no complications were identified.

**Table 3:** Baseline clinical characteristics of infants with IA in EOF and DOF groups after PSM.

Group		EOF (*n* = 179)	DOF (*n* = 319)	*p*
Diseases	Intestinal atresia (*n* = 23)	6	17	0.962
	Intestinal mass (*n* = 25)	10	15	
	Intestinal necrosis (*n* = 30)	11	19	
	Intestinal stenosis (*n* = 14)	7	7	
	Biliary disease (*n* = 157)	57	100	
	Stoma closure (*n* = 186)	65	121	
	Patent vitelline duct (*n* = 17)	6	11	
	Duodenal obstruction (*n* = 43)	16	27	
	Adhesional obstruction (*n* = 3)	1	2	
Sites of anastomosis	Duodenal anastomosis (*n* = 44)	16	28	0.821
	Jejunal anastomosis (*n* = 174)	63	111	
	Ileal anastomosis (*n* = 144)	52	92	
	Colonic anastomosis (*n* = 96)	37	59	
	Ileal-colonic anastomosis (*n* = 30)	11	29	
Laparoscope (yes/no, *n*)		35/144	52/267	0.359
Sex (male/female, *n*)		103/76	185/134	0.922
Age at surgery (d, Median [P25%, P75%])		91.0 [39.0, 193.0]	90.0 [43.0, 182.0]	0.638
Weight at surgery (kg, Median [P25%, P75%])		5.2 [4.0, 7.0]	5.2 [3.8, 7.0]	0.406
Weight/age at surgery (g/d, Median [P25%, P75%])		59.5 [35.2, 110.3]	59.6 [38.0, 104.5]	0.962
Pre-operative albumin (g/L, Median [P25%, P75%])		39.7 [36.0, 43.0]	40.1 [36.3, 43.3]	0.553
Pre-operative prealbumin (g/L, Median [P25%, P75%])		0.16 [0.11, 0.17]	0.14 [0.10, 0.17]	0.293

Mann-Whitney U tests and Pearson chi-square tests were used.

### 3.3. EOF complications in infants who underwent IA

The abdominal distension and vomiting rates in the EOF group were significantly higher than the DOF group (17.9% vs. 7.2%, *p* < 0.001, 7.8% vs. 2.5%, *p* = 0.006), respectively; however, no difference were detected in failing to reach the initial OF between the two groups (3.9% vs. 1.8%, *p* = 0.173). There were no differences in the incidence of diarrhea, hematochezia, and anastomotic leakage between the EOF and DOF groups in infants who underwent IA (all *p* > 0.05; Table 4).

**Table 4:** Complications in infants with intestinal anastomosis after EOF or DOF.

Group	EOF (*n* = 179)	DOF (*n* = 319)	*p*
**Abdominal distension (yes/no, *n*)	32/147	23/296	<0.001
*Vomit (yes/no, *n*)	14/165	8/311	0.006
Diarrhea (yes/no, *n*)	4/175	9/310	0.694
Hematochezia (yes/no, *n*)	1/178	2/317	0.925
Anastomotic leakage (yes/no, *n*)	0/179	1/318	0.454
Failure to initial OF (yes/no, *n*)	7/172	6/313	0.173

Pearson’s chi-square tests were used. *p < 0.05, **p < 0.001.

The time to total parenteral nutrition (TPN), PN, and postoperative hospital stay were significantly shorter in the EOF group than the DOF group in infants who underwent an IA [2.0 (1.0, 2.0) d vs. 5.0 (3.0, 6.0) d; 6.0 (5.0, 8.0) d vs. 8.0 96.0, 11.0] d; 10.0 (7.0, 14.0) d vs. 12.0 (9.0, 15.0) d, respectively; all *p* < 0.001. There were no differences with respect to weight, age, the weight-to-age ratio, and the serum albumin and prealbumin levels at the time of hospital discharge between the EOF and DOF groups (all *p* > 0.05; Table 5).

**Table 5:** Recovery of infants with intestinal anastomosis after EOF or DOF.

Groop	EOF (*n* = 179)	DOF (*n* = 319)	*p*
Weight at discharge (kg, Median [P25%, P75%])	5.4 [4.2, 7.2]	5.2 [3.8, 7.0]	0.190
Weight/age at discharge (g/d, Median [P25%, P75%])	61.0 [35.7, 108.7]	58.9 [38.3, 104.1]	0.819
Albumin at discharge (g/L, Median [P25%, P75%])	39.0 [35.1, 43.2]	39.5 [35.4, 42.9]	0.672
Prealbumin at discharge (g/L, Median [P25%, P75%])	0.15 [0.12, 0.19]	0.14 [0.11, 0.17]	0.088
**Time of TPN (d, Median [P25%, P75%])	2.0 [1.0, 2.0]	5.0 [3.0, 6.0]	<0.001
**Time of PN (d, Median [P25%, P75%])	6.0 [5.0, 8.0]	8.0 [6.0, 11.0]	<0.001
**Hospital stay (d, Median [P25%, P75%])	10.0 [7.0, 14.0]	12.0 [9.0, 15.0]	<0.001

Mann-Whitney U tests were used. **p < 0.001.

## 4. Discussion

### 4.1. Low EOF rate in infants following an IA procedure

In the present study we found that the total EOF rate in neonates and infants who underwent an IA was only 20.3%; however, there were reports that early oral feeding could be well tolerated in 71–86% of adult patients with IA (32, 33). Although no recommended EOF rate in infants has been reported, the EOF rate among infants in our study was low and was not acceptable. IA is a standard surgical procedure for correcting digestive diseases; however, there is no clear criterion regarding the initial time of OF after an IA in infants. ESPEN (26, 28) recommends oral enteral nutrition within a few hours after an IA. Nevertheless, OF should be implemented after an adequate assessment of the patient’s tolerance and the type of surgery. The main reason for this recommendation may be that EOF after intestinal surgery increased the risk of anastomotic leakage or postoperative ileus, which is a potentially life-threatening condition (34, 35).

In the present study, based on multivariate logistic regression, the sites of anastomosis and weight at the time of surgery were two independent factors associated with EOF, while the disease diagnosis, age at the time of surgery, and preoperative serum albumin and prealbumin levels did not demonstrate differences. The different results between univariate analysis and multivariate logistic regression showed that disease diagnosis, age at the time of surgery, and preoperative serum albumin and prealbumin levels were interactive factors that were influenced by the sites of anastomosis and weight at the time of surgery. The EOF rates following jejunal and colonic anastomoses were relatively higher than ileal-colonic anastomoses. The reason for this finding was unknown; however, concerns about anastomotic leakage may be the main reason (6). Most of the diseases in the jejunal and colonic anastomoses subgroups were biliary diseases and stoma closures of anorectal malformations, which may be associated with a similar diameter between the proximal and distal intestine and low risk for anastomotic leakage. In the current study it was shown that higher weight at the time of surgery may be associated with a higher rate of EOF, which may indicate that nutritional status is important when initiating EOF. Our study showed a low rate of EOF in every type of intestinal anastomosis, which highlighted the concern that exists about EOF among most pediatric surgeons. Evidence for initiating EOF after an IA procedure in infants was insufficient in a previous study (24) and a deep evaluation of the safety and efficacy of EOF is necessary with a large number of cases. This was a real-world study using PSM conducted in a relatively large number of patients to evaluate the safety and efficacy of EOF in infants following an IA.

### 4.2. A similar incidence of EOF complications compared to DOF in infants following an IA

In our study a higher incidence of abdominal distension and vomiting occurred in the EOF group, which indicated that some gastrointestinal complications were associated with EOF; however, the rate of failure to initial OF did not indicate a difference between the EOF and DOF groups, which demonstrated that the incidence of abdominal distension and vomiting in EOF were acceptable. Traditionally, it has been believed that DOF at least 4–5 days after an IA has a protective role at the anastomosis site; however, several studies have shown that this conclusion is incorrect (8, 9). Several studies have confirmed the safety of postoperative EOF compared to DOF in decreased or similar incidences of complications, such as vomiting, abdominal distention, and anastomotic leakage (7, 14, 15, 24). Prolonged fasting after an IA is poorly tolerated in infants and is more likely to cause problems than in older children and adults (24). DOF may increase the use of PN, which is associated with cholestasis in infants (36). In contrast, EOF relieves cholestasis and reduces the incidence of sepsis (23). The increased PN following DOF may be associated with indwelling venous catheters, trauma, length of hospital stay, and hospital costs, which are not acceptable according to ERAS (37–39). Therefore, it is necessary to prove the safety of EOF in infants who undergo an IA, and implement EOF it into practice. In our study the safety of EOF was widely evaluated, and abdominal distention, vomiting, and diarrhea were shown to be common complications of EOF in infants undergoing an IA, which is consistent with the literature (16). The complications in EOF were comparable to DOF, which may indicate the safety of EOF in infants.

In our study no higher rates of hematochezia, diarrhea, and anastomotic leakage were associated with EOF, which indicated good tolerance of EOF in infants who underwent an IA. Postoperative DOF further aggravates nutrient consumption and insulin resistance, which results in impaired immune function, delayed wound healing, decreased muscle strength, and increased complications (16), and is primarily associated with delayed gut development and metabolic diseases in neonates and infants (21, 22). EOF can stimulate the secretion of digestive juices, which may enhance the recovery of intestinal function (7, 26). In addition, animal experiments have shown that early OF promoted the synthesis of collagen in the anastomosis and affected fibroblasts to maintain the stability of the anastomosis (12, 13). In the present study there was no anastomotic leakage in the EOF group for any anastomosis type. EOF stimulates the release of intestinal hormones and triggers intestinal persistence, which also enhances recovery of intestinal function and reduces the occurrence of postoperative intestinal obstruction (23, 40). Therefore, in combination with the recommendations of the ESPEN Perioperative Nutrition Guidelines (26–28), EOF after an IA in infants may be safe.

### 4.3. EOF enhanced recovery after IA in infants

The present study showed that EOF reduced the need for PN and TPN with the postoperative nutritional status in the EOF group, which was similar to the DOF group in infants with IA. EOF is a common component of ERAS, which can reduce the psychological, physical stress, and trauma caused by surgery and promote fast postoperative recovery (37, 38). Amanollahi and Azizi (8) conducted a controlled clinical study 1 decade ago involving 67 children who underwent an IA and proved that EOF had significant advantages over DOF in reducing the first defecation time and postoperative hospital stay. The two most recent systematic reviews also confirmed that EOF promoted early bowel recovery and hospital discharge compared to DOF (7, 16). The reduced use of PN may help decrease cholestasis and sepsis (39). Some reports have indicated that EOF relieved cholestasis (41, 42). A prospective randomized controlled study in children undergoing enterostomy closure indicated that EOF reduced the risk of postoperative infections (14). These findings indicate that it might be beneficial to perform EOF after various gastrointestinal anastomosis site types in infants.

In this study the hospital stay was shortened by EOF for infants who underwent an IA, which may be attributed to the decreased use of PN. As a result of the rapid growth and development of infants, DOF or increasing the usage of PN delays gut development, reduced the regeneration of intestinal mucosal epithelial cells, and increased apoptosis, thus leading to mucosal atrophy, barrier function impairment, and eventually intestinal bacterial translocation and sepsis (21, 39); however, EOF has been shown to be beneficial in gut development, regeneration of the intestinal mucosal epithelium, maintenance of the integrity of the gut barrier, and prevention of bacterial translocation (43, 44), which promotes the recovery of intestinal function. These findings indicate that EOF following an IA is effective in infants.

## 5. Conclusion and limitations

The overall EOF rate in infants following an IA was low, and the sites of anastomosis and weight at age were two factors associated with EOF. Nevertheless, performing EOF in infants after an IA was safe and effective, reduced PN usage, shortened the hospital stay, and did not increase the severe complication rate.

The overall number of cases in this multicenter study was relatively large; however, the number of EOF cases was insufficient. In addition, the neonatal period and infancy are only short-term stages in childhood, and safe and effective EOF after an IA in infants cannot be extended to childhood. Moreover, this study was not a randomized controlled study, which may be associated with incontrollable bias. A more extensive multicenter, prospective, randomized controlled study should be conducted in the future.

## Data availability statement

The original contributions presented in the study are included in the article/supplementary material, further inquiries can be directed to the corresponding authors.

## Ethics statement

The studies involving human participants were reviewed and approved by Institutional Ethics Committee of the Children’s Hospital of Nanjing Medical University (approval number: 202004018-1). Written informed consent to participate in this study was provided by the participants’ legal guardian/next of kin.

## Author contributions

WT and QG contributed to conception and design of the study. XS and CL organized the database. CL performed statistical analysis and wrote the first draft of the manuscript. All authors contributed to the article and approved the submitted version.

## Funding

This study was funded by the Top of Jiangsu Provincial Health Committee’s Six One Project, China (LGY2020019) and the General Project of Nanjing Health Commission (YKK20121).

## Conflict of interest

The authors declare that the research was conducted in the absence of any commercial or financial relationships that could be construed as a potential conflict of interest.

## Publisher’s note

All claims expressed in this article are solely those of the authors and do not necessarily represent those of their affiliated organizations, or those of the publisher, the editors and the reviewers. Any product that may be evaluated in this article, or claim that may be made by its manufacturer, is not guaranteed or endorsed by the publisher.

## Abbreviations

IA: Intestinal anastomosis
OF: Oral feeding
EOF: Early oral feeding
DOF: Delayed oral feeding
PSM: Propensity score matching
